# Scientometric study of the effects of exposure to non-ionizing electromagnetic fields on fertility: A contribution to understanding the reasons of partial failure

**DOI:** 10.1371/journal.pone.0187890

**Published:** 2017-12-06

**Authors:** Nicola Bernabò, Rosa Ciccarelli, Luana Greco, Alessandra Ordinelli, Mauro Mattioli, Barbara Barboni

**Affiliations:** Faculty of Bioscience and Agro-Food and Environmental Technology, University of Teramo, Teramo, Italy; Institut Català de Paleoecologia Humana i Evolució Social (IPHES), SPAIN

## Abstract

The exposure to Non-Ionizing-Electromagnetic Fields (NI-EMFs) is often indicated as a cofactor responsible for the fertility reduction, which has been described in recent years. Despite the great interest in this topic and the research effort in exploring it, to date, there are no reliable data. Therefore, we carried out a scientometric analysis of the scientific literature published in peer reviewed Journals concerning this topic to better understand the reasons of this partial failure. To this aim, we identified and analysed 104 papers, published in last 26 years in peer-reviewed Journals, present in ISI Web of Knowledge Core Collection. Then, we analysed the impact of the Journals in which the papers were published as well as that of the single papers, the paper citation dynamics, the keywords citation busts, the geographical localization of citations and the co-authorship dynamics of the Authors. As a result, we found that different animal models (rodent, rabbit, guinea pig, and swine) and different experimental approaches (epidemiological vs. experimental studies) have the same impact, highlighting the lack of universally adopted standard in research activity. The analysis of the temporal trend in keywords and the high differences in citations between the different countries (also in those belonging to the same geographical and socio-economical area) pointed out the difficulties in approaching this branch of study. Lastly, it was evident that the Authors did not behave as a connected community, but as unconnected clusters of very small size. In conclusion, based on the results of our analysis, we think that important efforts must be undertaken to adopt more standardized models and to improve the research quality and the information exchange within the scientific community, with the aim of improving the reliability and usefulness of the results of research regarding the effect of NI-EMFs on fertility.

## Introduction

Important international Agencies claim that in recent years human fertility is decreasing in developed countries [1,2]. To date, there are not conclusive certainties about this phenomenon and its causes are still obscure. During the years, different possible factors have been proposed to contribute to the accumulation of infertility risk factors. In particular, different conditions related to social changes have been taken into account as well as to lifestyle [3,4], such as tobacco [5–7]and marijuana smoking [8–10], alcohol [11,12], medication [13], caffeine [14], and the exposure to pesticides, solvents [15,16]and electromagnetic fields EMFs [17–19]. This last case, in particular, consists of electromagnetic waves characterized by frequency *f*, wavelength λ, and photon energy *E*. The frequency is inversely proportional to the wavelength and is directly proportional to the photon energy as described by Planck’s law:

$$E=\frac{hc}{\lambda }$$

where: *h* = 6.62606896(33)×10−34 J·s = 4.13566733(10)×10−15 eV is Planck's constant.

The range of all possible frequencies is called “the electromagnetic spectrum” and ranges from 0Hz (static magnetic fields, SMFs), to 2.4×10^23^Hz (γ rays). After billions of years of coexistence among biological organisms with EMFs of natural origin, in the last century the explosion of human technological activity has dramatically increased the presence in the biosphere of non ionizing radiations (NIR), i.e. EMFs whose energy is lower than the ionization energy of hydrogen (14 eV). In particular, the exposure to specific classes of NI-EMFs, such as static magnetic fields (SMFs), extremely low frequency electromagnetic fields (ELF-EMFs), radiofrequencies(RFs) and microwaves (MWs), had enormously increased.

SMFs are generated during the medical imaging procedures of Nuclear Magnetic Resonance Imaging (NMRI), when the patients are exposed for 30–60 min to three different EMFs: field gradients, radiofrequencies (RFs), and the static magnetic field (SMF). In particular, the SMF has intensities that usually range from 1–7 T, i.e. hundreds of thousands times stronger than those present in nature (the geomagnetic field on the Earth's surface ranges from 25 to 65μT).

ELF-EMFs are defined as the electric and magnetic fields in the frequency range >0 to 100 kHz, the most important of which are of 50 and 60Hz, i.e. the frequencies generated by the production, transport and fruition of electricity in Europe and the USA, respectively.

RFs and MWs are used in Information and Communication Technology (ICT), for instance in cell phone, Wi-Fi, and Bluetooth protocols and in specific working condition (i.e. microwave welding).

Consequently, humans are continuously exposed to EMFs in public places, houses, schools, workplaces, and hospitals, thus originating in the public opinion and scientific community important concerns about their possible negative effects on health. To date, the International Agency for Research on Cancer, IARC, based on epidemiological and experimental, in vitro and in vivo studies, classified SMFs in group 3 (“not classifiable as to their carcinogenicity to humans”), while ELF-EMFs and RFs are classified as 2b (“possibly carcinogenic to humans”)[20]. As regards the possible toxic effect of EMFs on fertility unfortunately, the data now available, are not conclusive, thus it is impossible for scientists to offer the public opinion and decision-making organisms, reliable recommendations.

Here we carried out a scientometric analysis of the scientific literature, published in peer reviewed Journals, concerning this important issue with the aim of taking an updated picture of this branch of research. To this aim, in keeping with a validated approach already adopted by our [21] and other groups [22], we decided to carry out the quantitative assessment of several parameters known to be related with the scientometric evaluation of research activity. In particular, we analysed qualitative and quantitative parameters related to the papers and Journals that contain them, on experimental models and analytical approaches used, and on the authors’ co-authorship dynamics. We hope that the data we provide will be helpful to identify a new strategy in planning future research activity and in improving the strength of research results.

## Materials and methods

### Data collection

As data source, we used the papers published between, January 1^st^, 1996 and May 31^th^, 2016 contained in Web of Science Core Collection (WoS) [23]. To select the paper used in this study, we used the Advanced Search Function of WoS, that uses field tags, Boolean operators, and query sets to create specific queries. For example, we used the following syntax:

TS=(topic1)ANDTS=(topic2)

Where: TS is the topic

                AND is the Boolean operator

In our queries, we used as topic 1 “fertility” combined with the following key words as topic 2:“Static Magnetic Fields”, “Electromagnetic Fields”, “Extremely Low-Frequency Electromagnetic Fields”, “Radiofrequency Electromagnetic Fields”, “Wi-Fi”, “Bluetooth”, “Microwave”.

We classified all the papers based on the biological model studied (human, rat, mouse, rabbit, guinea pig, swine), on the spectrum of EMF considered (SMF, ELF, RF) and on the experimental approach (epidemiological studies, in vitro or in vivo experiments). We calculated the number of paper citations per year in order to measure publications impact, and we assessed the impact factor (IF) and the 5 year IF of each journal to measure journals impact. Since these values change along the years we used, were possible, the data referred to 2015 and to 2015–2011 period, respectively. Otherwise, we used the most recent available data.

### Analysis of ISI key words and geographic distribution of EMFs papers citations

The data related to the selected papers were processed for temporal and geospatial analysis by Sci^2^ Tool (Sci^2^ Team)[24]. We generated a temporal visualization of the burst of ISI keywords used in the papers, and a choropleth map that shows the geographic distribution of the selected papers distinguished by shades of colour for each Country, proportional to the number of citations.

### Map of science

To explore the closeness of scientific disciplines related to the study of the effect of EMFs on fertility we realized, by using Sci^2^ software, a map of science. It is a visual representation of a network of 554 subdisciplines (represented as nodes), that are aggregated to 13 main disciplines of science. Mapped subdisciplines are shown by size, related to numbers for journals and colours for disciplines.

### Co-authorship network

To study the co-authorship dynamics of the Authors, we used an approach based on social networks, representing them as nodes of a network and, when two or more authors share a publication, they are linked by an edge. The open-source software Cytoscape 2.8.3 [25] has been, for network creation, visualization and analysis, carried out considering the networks as undirected. To study the topology of the networks obtained, in keeping with a previous work [21], we automatically computed the main topological parameters listed above, using:

*Number of nodes*: It is the total number of Authors involved.
*Number of edges*: It is the total number of interactions found.
*Connected Components*: It is the number of networks in which any two vertices are connected to each other by links, and which is connected to no additional vertices in the network.
*Clustering coefficient*: It is calculated as *C*I = 2*n*I/*k*I(*k*I–1), where *n*I is the number of links connecting the *k*I neighbours of node I to each other. It is the measure of how the nodes tend to create clusters.
*Network diameter*: It is the longest of all the calculated shortest paths in a network.
*Characteristic path length*: It is the expected distance between two connected nodes.
*Averaged number of neighbours*: It is the mean number of connections of each node.
*Node degree*: It is the number of interactions for each node.
*Node degree distribution*: It represents the probability that a selected node has *k* links.
*γ*: It is the exponent of node degree equation.
*R*^*2*^: It is the coefficient of determination of node degree vs. number of nodes, on logarithmized data.

The statistical analysis of network organization (the so called “topology”) was used to take some inferences about the pattern of social behavior of Authors.

### Data analysis

All the bibliometric and citational data related to the selected papers were checked for normality using the D’Agostino and Pearson normality tests. As they are not parametrical, we used the appropriate descriptive and inferential techniques, such as the Kruskal-Wallis or Mann-Whithey test depending on the needs, and the data are shown as median (25°percentile– 75°percentile).

## Results and discussion

It has been suggested that the exposure of humans to non-ionizing electromagnetic fields could be a contributory cause of the decrease in fertility. Here, we conducted a scientometric analysis of the literature concerning this topic, with the aim of taking an updated picture of the scientific production and of its impact on the scientific community. In addition, we studied the co-authorship dynamics of the authors involved in this field.

As first, we have found that the number of papers available on Web of Science Core Collection is relatively low. We found 107papers concerning the effects of NI-EMFs on mammalian fertility. Since three of them have been discarded (two are referred to a non-mammalian model, D. Melanogaster, and the third has been retired), for the further analysis we considered 104 papers (see Table 1).

**Table 1:** List of selected papers.

WOS AccessionNumber	ExperimentalApproach	Biological Model	Year of Publication	Citations per Year	IF	5 years IF
STATIC MAGNETIC FIELDS–SMFs						
A1996VX74000020	Experimental	mouse	1996	1.150	8.443	9.098
000084818100004	Experimental	mouse	2000	1.188	1.583	1.788
A1994QF41100007	Experimental	rat	1994	3.727	2.141 (2004)
000327353100011	Experimental	swine	2013	0.000	1.208	1.162
000075324200059	epidemiological	human	1998	0.333	4.621	4.635
EXTREMELY LOW FREQUENCY ELECTROMAGNETIC FIELDS—ELF EMFs						
000173700900009	experimental	mouse	2002	2.929	1.583	1.788
000220310000005	experimental	mouse	2004	5.583	2.644	2.579
000267594400007	experimental	mouse	2009	0.714	0.188	0.342
000262310600009	experimental	mouse	2009	0.000	1.583	1.788
000279430400007	experimental	mouse	2010	1.000	1.208	1.162
000296459500006	experimental	mouse	2011	1.400	1.208	1.162
000329505400003	experimental	mouse	2014	2.000	1.583	1.788
000341343800006	experimental	mouse	2014	0.500	1.165	1.265
000362048600015	experimental	mouse	2015	0.000	1.275	1.339
000169580200007	experimental	rat	2001	3.267	1.583	1.788
000230823300010	experimental	rat	2005	2.727	2.644	2.579
000234773500007	experimental	rat	2006	4.400	1.583	1.788
000256627200006	experimental	rat	2008	1.500	1.208	1.162
000265656700002	experimental	rat	2009	0.000	1.603	1.369
000267633500001	experimental	rat	2009	1.000	1.688	1.786
000270194600007	experimental	rat	2009	1.000	1.688	1.786
000357431400014	experimental	rat	2010	0.500	1.441	1.474
000290290800004	experimental	rat	2011	3.400	2.722	2.848
000303760700004	experimental	rat	2011	0.000	0.839 (2011)	0.875
000299632500005	experimental	rat	2012	1.750	2.722	2.848
000329867500007	experimental	rat	2013	0.000	1.779	1.933
000327607800006	experimental	rat	2014	3.000	1.583	1.788
000335765200007	experimental	rat	2014	0.500	1.208	1.162
000268931800013	experimental	rabbit	2009	0.000	1.276	1.305
000277962000016	experimental	swine	2010	3.000	1.838	2.056
000301415200008	experimental	human	2011	0.000	0.366	0.532
A1993MN54400002	epidemiological	human	1993	2.217	2.85	3.401
000079213100006	epidemiological	human	1999	1.294	3.745	3.49
000073892200004	Review	human	1998	2.333	5.261	5.956
000246125100013	Review	human	2007	0.000	1.128	1.639
000246125100014	Review	human	2007	0.000	1.128	1.639
RADIOFREQUENCIES—RFs						
000239219600018	experimental	Mouse	2006	3.800	2.85	3.401
000262187700009	experimental	Mouse	2009	4.286	3.022	3.072
000334273900001	experimental	Mouse	2014	1.500	2.949	3.167
000072701500004	experimental	Rat	1998	0.667	0.31 (2003)	
000229298500008	experimental	Rat	2005	6.182	0.562	0.639
000250192800028	experimental	Rat	2007	6.667	4.426	4.333
000251984000005	experimental	Rat	2008	5.125	2.219	2.399
000265889100020	experimental	Rat	2009	0.286	0.365	0.359
000267705200010	experimental	Rat	2009	8.714	1.328	1.444
000270200400007	experimental	Rat	2009	3.714	1.68	1.513
000283681600004	experimental	Rat	2010	2.500	0.562	0.639
000269760500005	experimental	Rat	2011	0.000	0.812	0.816
000288010900055	experimental	Rat	2011	0.000	4.426	4.333
000290227000013	experimental	Rat	2011	7.800	1.606	1.855
000290292700002	experimental	Rat	2011	1.400	0.343	0.399
000293863900007	experimental	Rat	2011	4.800	1.204	1.644
000294436800020	experimental	Rat	2011	0.000	1.328	1.444
000306864900078	experimental	Rat	2012	2.500	1.504	1.571
000307588400005	experimental	Rat	2012	1.500	1.208	1.162
000307588400006	experimental	Rat	2012	1.500	1.208	1.162
000315633800002	experimental	Rat	2013	1.667	1.779	1.933
000316307000001	experimental	Rat	2013	3.333	2.85	3.401
000317837600026	experimental	Rat	2013	3.667	1.17	1.279
000323611200014	experimental	Rat	2013	2.000	1.208	1.162
000327569100022	experimental	Rat	2013	4.333	2.85	3.401
000330046300005	experimental	Rat	2013	0.333	0.971	1.019
000331338700024	experimental	Rat	2014	0.500	3.25	3.449
000335765200001	experimental	Rat	2014	2.000	1.208	1.162
000340868200025	experimental	Rat	2014	1.500	2.309	2.649
000298926600026	experimental	Rat	2015	2.000	0.539	0.564
000338399500005	experimental	Rat	2015	0.000	1.275	1.339
000360029900007	experimental	Rat	2015	0.000	1.127	0.87
000289040800015	experimental	rabbit	2009	0.000	0.372	0.413
000349768200005	experimental	rabbit	2015	0.000	1.208	1.162
000361005400006	experimental	guineapigs	2009	0.143	1.0	0.938
000268637600002	experimental	human	2009	19.286	3.057	3.535
000270616100029	experimental	human	2009	15.429	4.426	4.333
000280984100005	experimental	human	2010	2.833	3.022	3.072
000286110000004	experimental	human	2011	4.600	3.695	3.265
000298367600011	experimental	human	2012	10.250	4.426	4.333
000323180300007	experimental	human	2013	1.000	2.429	2.208
000231271000007	epidemiological	human	2005	5.091	3.057	3.535
000255254900009	epidemiological	human	2008	4.375	7.105	6.434
000256952300003	epidemiological	human	2008	1.500	1.583	1.788
000295174100005	epidemiological	human	2011	5.400	1.441	1.474
000296935100014	epidemiological	human	2011	1.000	2.85	3.401
000357481700007	epidemiological	human	2015	0.000	1.214
000360655700014	epidemiological	human	2015	0.000	2.796	2.722
000182310000001	review	human	2003	6.769	1.057	1.227
000234832700002	review	human	2006	2.500	0.891	0.896
000246296500013	review	human	2007	0.222	0.891	0.896
000247917700025	review	human	2007	8.444	0.895	1.215
000254304100001	review	human	2008	6.500	3.98	4.002
000262709100021	review	human	2009	3.143	2.796	2.722
000268794800001	review	human	2009	2.429	1.165	1.265
000315161000002	review	human	2012	4.500	1.627	1.789
000338038400003	review/meta analysis	human	2014	2.000	2.515	2.515
000339693200011	review/meta analysis	human	2014	8.000	5.929	6.604
000078267000006	review	All	1998	0.944	0.925	
000300365100002	review	All	2011	5.800	0.871	1.105
000302070800002	review	All	2012	0.000	1.858	2.057
ELECTROMAGNETIC FIELDS (VARIOUS)						
000296459500003	experimental	rat	2011	0.800	1.208	1.162
000312237600006	experimental	rat	2012	1.750	2.227	2.243
000084136600016	epidemiological	human	1999	0.941	1.627	1.99
000232423000004	epidemiological	human	2005	2.000	5.036	5.471
000273802000011	epidemiological	human	2010	4.333	2.85	3.401
000173332300008	review	human	2001	3.133	1.041	1.352
A1992JL95500001	review	vertebrates	1992	2.917	3.817	3.967

The first part of our study was aimed to assess the impact of different animal models, experimental approaches (epidemiological study, in vitro or in vivo experiments), and classes of EMFs in research activity. As indicators, we used the number of papers published and the number of citations per year of each paper [21]. We found that the overall distribution of this parameter was represented by the following equation: y = 208.2 x^-1.869^ (R^2^ = 0.9011) which is in agreement with Bradford’s Law [26].About one third of the papers are referred to humans (35/104), while the most used animal model is rat (44.2%; 46/104) followed by mouse (13.5%; 14/104), rabbit (2.9%; 3/104), swine (1.9%; 2/104), and guinea pig (1%; 1/104). In our opinion, this finding is very interesting because it highlights that the most used animal models are rodents and rabbits (60.6% of papers). In these animals, the exposure to EMFs necessarily interests the whole body (usually they are exposed directly within the cages), thus it is impossible to discriminate the real reproductive effects from possible neuro-endocrine interferences, which constitute an important limit in interpreting the experimental data. The adoption of large animal models could be useful to overcome this limit, indeed in this context, it is possible to realize the exposure of the reproductive system without affecting the other endocrine or nervous structures [27].

When comparing the papers for differences in term of citations per year, depending on the animal model, we did not find significant differences [human 2.4 (0.95–4.85); rat 1.8 (0.5–3.38); mouse 1.3 (0.78–2.68), rabbit 0; swine 1.5 (0.75–2.25); guinea pig (0.1 (0.1–0.1); p>0.05, Kruskal-Wallis test].

As regard the EMF typology studied, we found that most of the papers were addressed to study the effects of RFs (58.7%; 61/104), followed by ELFs (29.8; 31/104) and SMFs (4; 3.8%). In 3 cases (2.9%) different classes of fields were analysed. The analysis of the number of citations per year confirms the higher interest in the study of RFs when compared to ELF [2.4 (0.7–4.8) vs. 1.0 (0–2.5) citations per year respectively, p<0.05, Mann-Whitney u test] and the relatively low impact of papers referred to SMFs [0.75 (0.23–1.2) citations per year].

This finding is justified by the increasing interest in the study of possible health effect of fields employed in ICT, whose exposure is exponentially increasing in recent years. Not surprisingly, the median age of papers is 5 years for those referred to RFs and 7 years for those referred to ELFs. Very interesting is the scarce number of papers on the effects of SMFs and their age (in median 17 years), which is in contrast the dramatic increase in the number of patients and workers exposed with different modalities to these fields.

Ultimately, most papers are experimental studies (70.9%; 73/104), 17.3% (18/104) are reviews and 12.5% (13/104) are epidemiological surveys. This finding is consistent with the idea that the use of animal models is essential in doing research in this field. In terms of citation per year, we did not find statistically significant difference among these different approaches [epidemiological studies 1.5 (0.9–4.3), experimental studies 1.5 (0.5–3.4), reviews 2.7 (1.17–5.48), p> 0.05, Kruskal-Wallis test].

**Fig 1:**
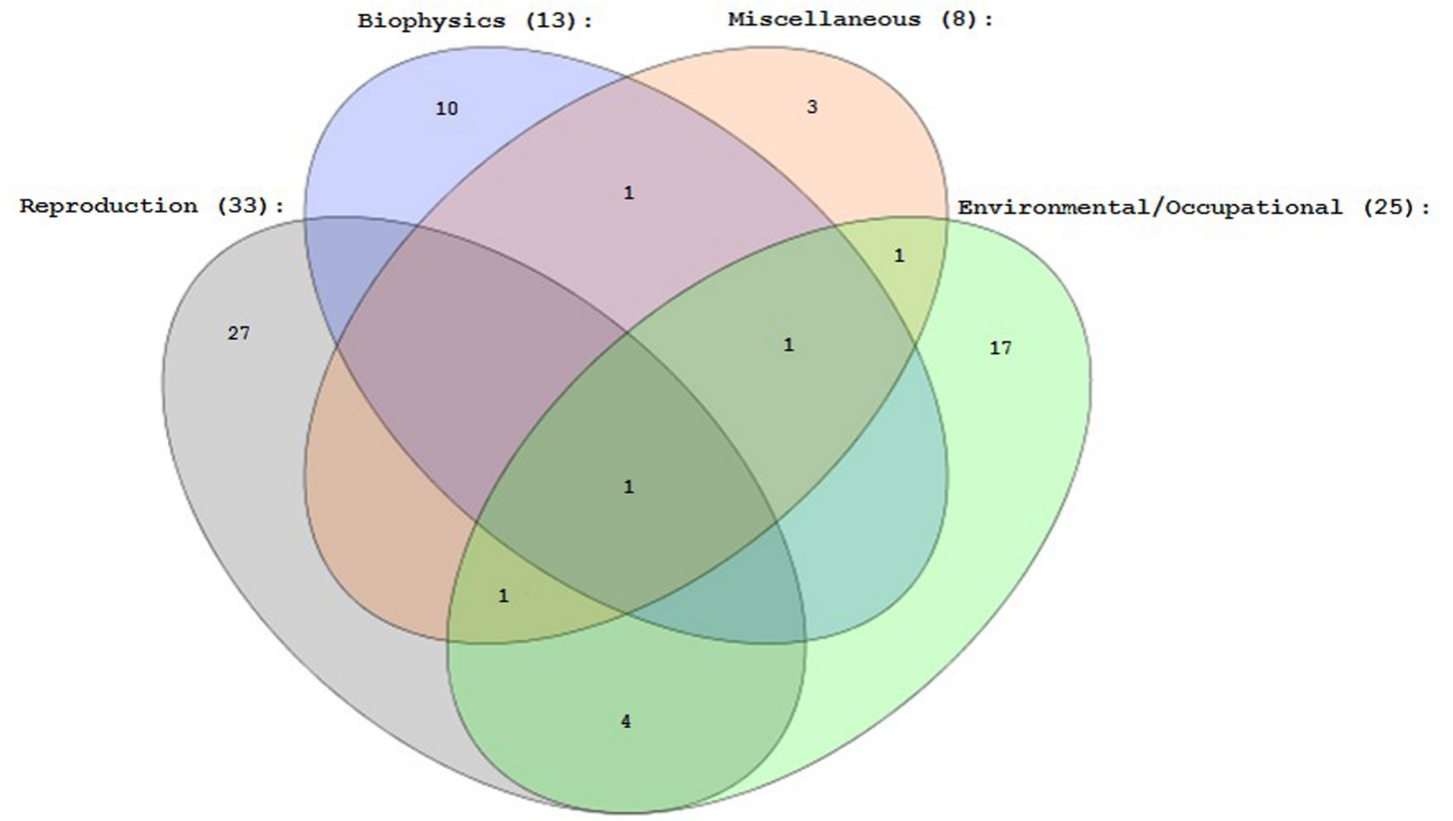
Venn’s diagram showing the number of Journals listed in different thematic areas (“Reproduction”, “Biophysics”, “Environmental/occupational toxicology”, “Miscellaneous”).

We classified the Journals in which the papers had been published in four thematic areas: “Biophysics”, “Reproduction”, “Environmental/Occupational toxicology”, “Miscellaneous”. Each Journal was listed in one or more of these classes, and we carried out an analysis using the set theory. Most papers were published in “Reproduction” Journals (33) then, in “Environmental/occupational toxicology” (25), in “Biophysics” (1), and in “Miscellaneous” (8), as reported in Fig 1.

This finding is very important, because in studying the effect of EMFs on biological samples the correct methodological approach in design and realization of EMF source has a key role. These Journals, perhaps, in the peer-review process and in the editorial choice, give less guaranties of the correctness of these aspects, underlining the importance of the biological aspect of the problem compared to the physical and engineering set up. Unfortunately, there are no Journals specifically devoted to the study of biophysics in reproductive cells, thus it is very hard to bring together the expertise of different specialists, with potential detrimental effects on the quality of science.

**Fig 2:**
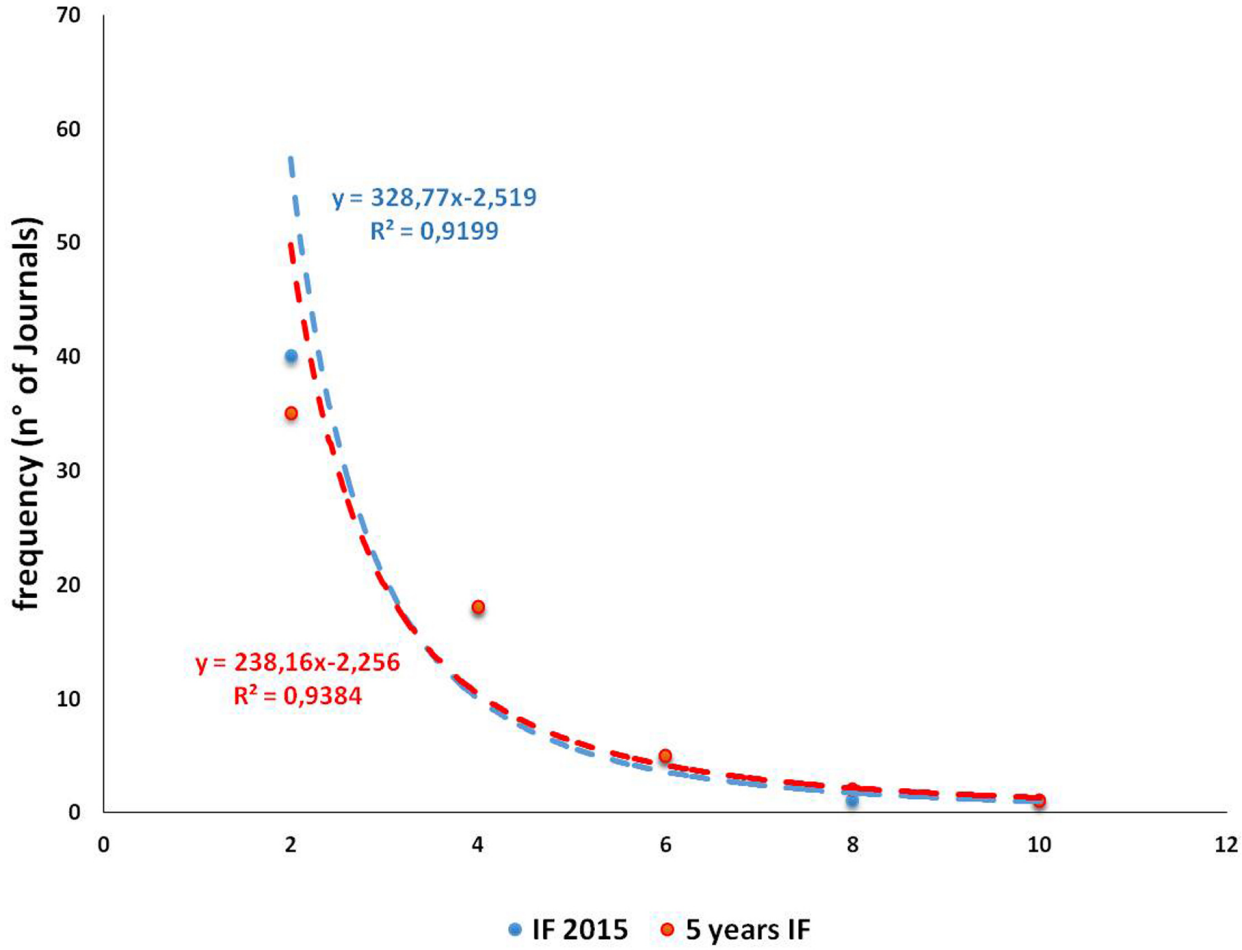
Graph showing the frequency of IF and 5 year IF in selected papers.

To complete this analysis, we assessed quantitative and qualitative parameters for the Journals. A higher number of papers was published in Electromagnetic Biology and Medicine (11 papers, 10.4%), Bioelectromagnetics (8 papers, 7.5%), Reproductive Toxicology (6 papers, 5.7%) and Fertility and Sterility (4 papers, 3.8%). We assessed the IF and the 5 year IF of the Journals: conscious of the limits of these parameters[21,28] we intended them as indicators of Journal impact and not as indicator of Journal quality. In both cases, the frequency distribution followed an exponential law (IF: y = 328.8x^-2.519^, R^2^ = 0.928; 5 year IF: y = 238.2 x^-2.256^, R^2^ = 0.938), as expected (see Fig 2).

**Fig 3:**
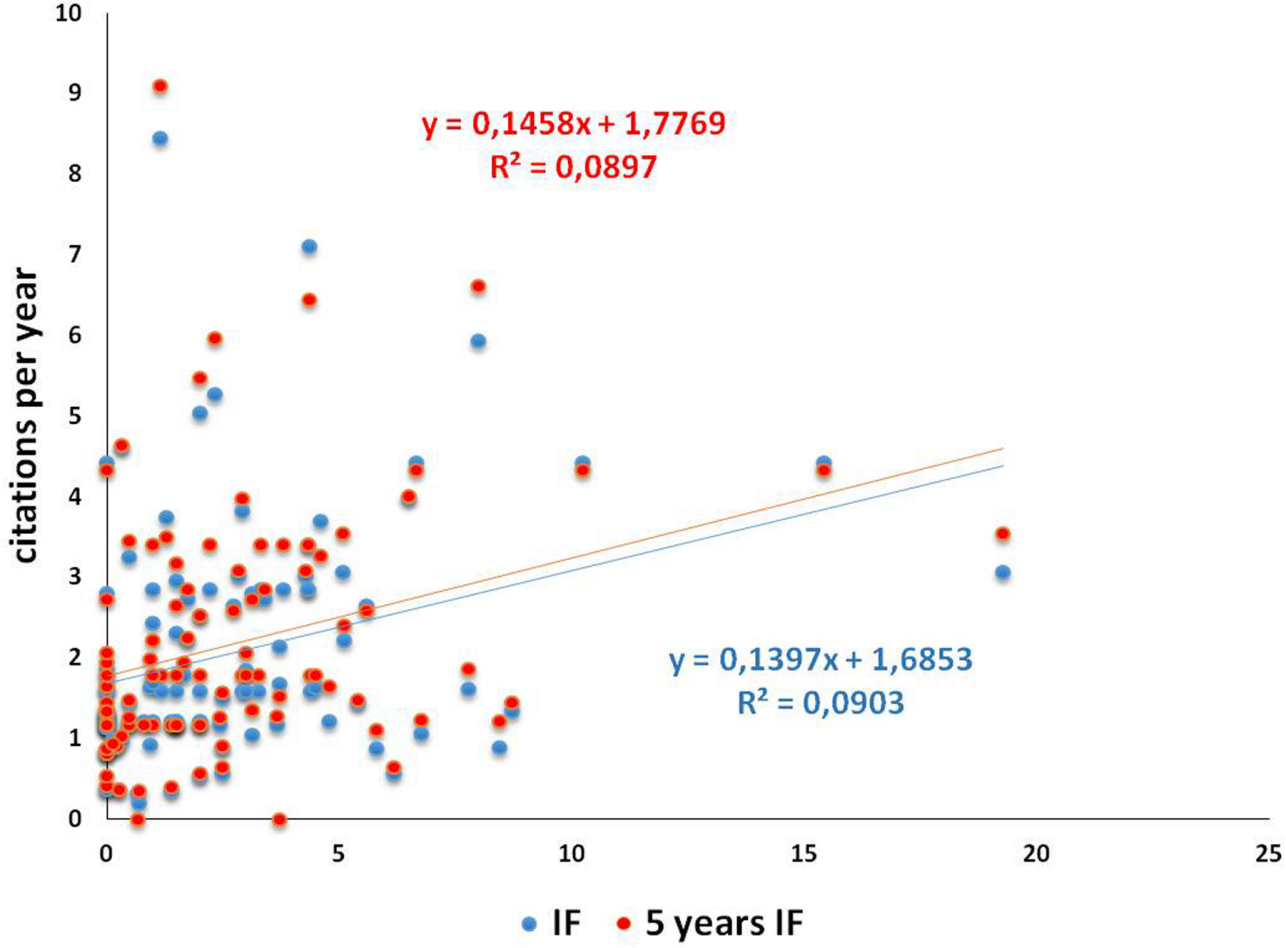
Graph showing the correlation between citations per year and IF or 5 year IF in selected papers.

The values of 25° percentile, median, and 75° percentile were: IF 1.606 (1.041–2.796) and 5 year IF 1.788 (1.215–3.072), and the maximum and minimum were 0.188–8.443 and 0.342–9.098, respectively. Referring to 5 year IF (more stable than IF) the Journals with the highest value (over the 75° percentile) are mainly related to the Environmental/occupational toxicology (Environmental Health Perspectives 9.089; Environment International 6.604; European Journal of Epidemiology 6.434; Mutation Research-Reviews in Mutation Research 5.956; American Journal of Epidemiology 5.471; International Journal of Hygiene and Environmental Health 4.002; Toxicology 3.967; Occupational and Environmental Medicine 3.490). Following are those related to Reproduction (Human Reproduction 4.635; fertility and Sterility 4.333, International Journal of Andrology 3.265), finally those of general interest, classified as Miscellaneous (PLoS One 3.535; Free Radical Research 3.167). Journal of Magnetic Resonance Imaging (3.449) was classified in Biophysics and Reproductive Toxicology (3.401) is classified both in Environmental/occupational toxicology and in Reproduction. The number of citations per year wasn't related either to the IF or to 5 year IF of the Journal in which the paper was published *(r* = 0.301 and *r* = 0.302, respectively) (Fig 3).

From this data analysis, it emerges that the Authors’ choice of Journal in terms of thematic area, the impact of Journals (IF and 5 year IF) and the impact of the single paper (measured in number of citations per year) does not respond to a well-defined pattern and it does not display an easily predictable behaviour. In our opinion, this makes the univocal fruition of research products more complex for readers and other scientists.

We analysed the ISI key words cited in the papers to identify the most important topics addressed, with particular regard to the time window in which they have been approached. As shown in Table 2, it is possible to identify a specific trend in the evolution of interests[21].

**Table 2:** List of citation bursts of ISI keywords in paperspublished in peer- reviewed Journals related to the effect of EMFs on fertility.

Class	ISI key word	Weight	Start	End
Generic keywords	fertility	2,718	2008	2009
	exposure	2,535	2006	2007
	in vitro	4,318	2011	2013
Processes	Lipid peroxidation	1,909	2012	2015
	oxidative stress	2,237	2012	2016
	Single strand	1,669	2005	2008
	melatonin	1,765	2005	2007
EMF source	Magnetic field	1,684	2012	2013
	video display terminals	1,933	1992	1998
	Microwave exposure	1,684	2012	2013
	60Hz	1,651	2012	2016
	Cellular phones	1,761	2012	2013
	Mobile phone radiation	1,782	2013	2016
	Mobile phones	1,837	2011	2016
Male fertility	Semen quality	1,293	1996	2008
	adult male	2,389	2008	2010
	Male fertility	2,826	2010	2011
	spermatogenesis	1,716	2012	2012
	Human spermatozoa	1,193	2012	2016
	Male infertility	4,476	2013	2016
Female fertility	Fetal development	1,829	1998	2005
	Spontaneous abortion	1,933	1992	1998

Class = phenomenon to which the keywords are referred. ISI keyword = keyword adopted by ISI system to classify the paper. Weight: intensity keyword of use of. Start = starting year of citation burst. End = end year of citation burst.

In the nineties of the last century, researchers attention was focused on the investigation of possible negative effects of exposure to video terminals, while more recently it has been directed towards studying the effects of exposure to mobile phone radiations. This finding highlights an interesting characteristic of the study of developing technology impact rapidly on human health. Sometimes the evolution of technologies is so fast that, on one hand, there is the risk that the answers regarding the effects of exposure to a specific EMF sources will arrive when the originating technology is obsolete, and, on the other hand, new technologies will rapidly diffuse before they are adequately studied. The key words referred to the molecular or physio-pathological determinants of the interaction between EMFs and biological systems are focused on oxidative stress, DNA damage, and melatonin. All these topics are closely related to the reproductive activity and represent potential targets of EMFs. In particular, the first two are of great interest because of their involvement in a myriad of biochemical pathways. The generation of ROS and their interaction with lipids and nucleic acids are reported to be involved in several pathological conditions, such as varicocele [29,30], exposure to heavy metals [31], carbon nanotubes [32], environmental toxicants [33], tobacco smoke [34,35] or simply aging [36]. This strengthens the idea that EMFs are co-stressors also involved in multifactorial pathogenic processes, instead of the concept of their role in causing pathologies. In the light of this consideration, the study of risk factor accumulation becomes very important as well as that of environmental pollution in general. It is very interesting, in addition, to note that the most of the biological events under study are related to male fertility, while on the side of female reproduction, only fetal development and spontaneous abortion have been considered by researchers. This could be due to the easier availability of male gametes, and to the difficulty in studying the effect of a cofactor in the context of female reproductive activity, which involves multi-organs and multi-system functions. This lack still represent**s** a challenge for scientists involved in the study of EMF effects on health as well as on female fertility.

**Fig 4:**
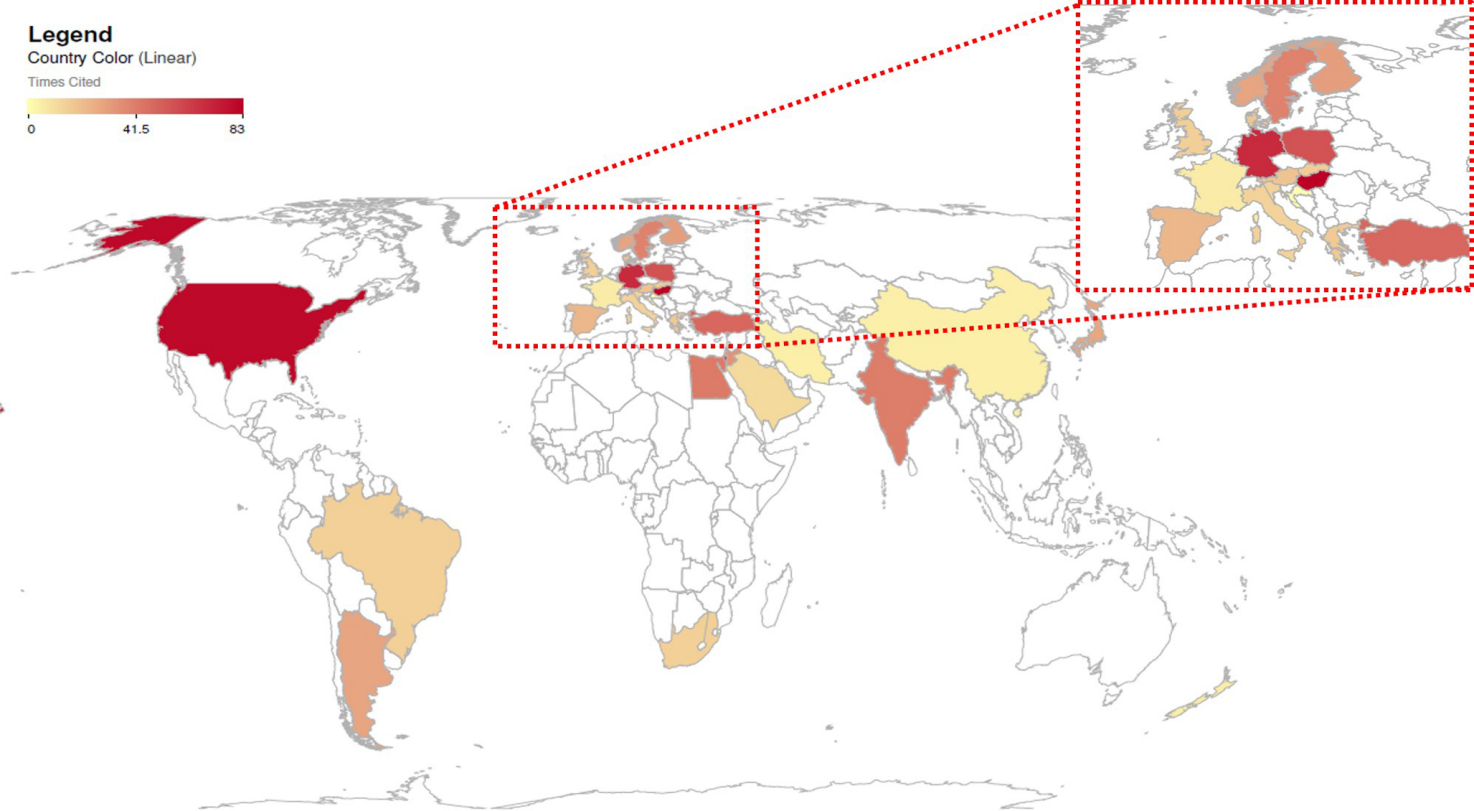
Geolocalization of scientific paper citations published in peer- reviewed Journals related to the effect of EMFs on fertility. The geographic distribution of the selected scientific papersis here relatedto the colour of each Country proportional to the citation number.

To study the contribution of different Countries and Geographical area on this kind of study, we carried out the georeferentiation of the citations of the examined. As a result, we found the data shown in Fig 4.

As it is evident, the developed Countries are characterized by a higher parameter, with the leadership of the USA and Europe. This datum is not per se surprising, but it provides the opportunity for two important considerations.

1. as seen in other scientific fields related to reproduction [21], several developing countries are excluded from research activity on such important issues. Here, it is interesting to note that China which is experiencing an amazing diffusion of technology and, consequently, exposure of humans to EMFs, seems to be scarcely active in research on possible negative consequences on fertility. On the contrary, India has a noticeable activity on this field.
2. Single European Countries display highly different behaviours. This is an interesting finding, because they have a similar technological development and are subjected to the same sovra-national policy of research funding. In the EU, the most important program for research funding is Horizon 2020. It is main as a top-down program, in which the priorities of funding have been a priori decided by the EU. Remarkably, here, the research funding on ICT has a central role, as stated by the EU, that claims that “ICT brings unique responses to society's challenges such as the growing needs for sustainable healthcare and aging well, for better security and privacy, for a lower carbon economy and for intelligent transport”[37], but there are not specific funding lines for the study of effects of EMF on health.
  In addition, the research funding policy on reproduction and reproduction-related issues in the EU changes according to the country[21,38]. This is due to the different scientific and regulatory traditions among the European countries and to the different weight of involved stakeholders (public opinion, patients associations, companies, etc.).

**Fig 5:**
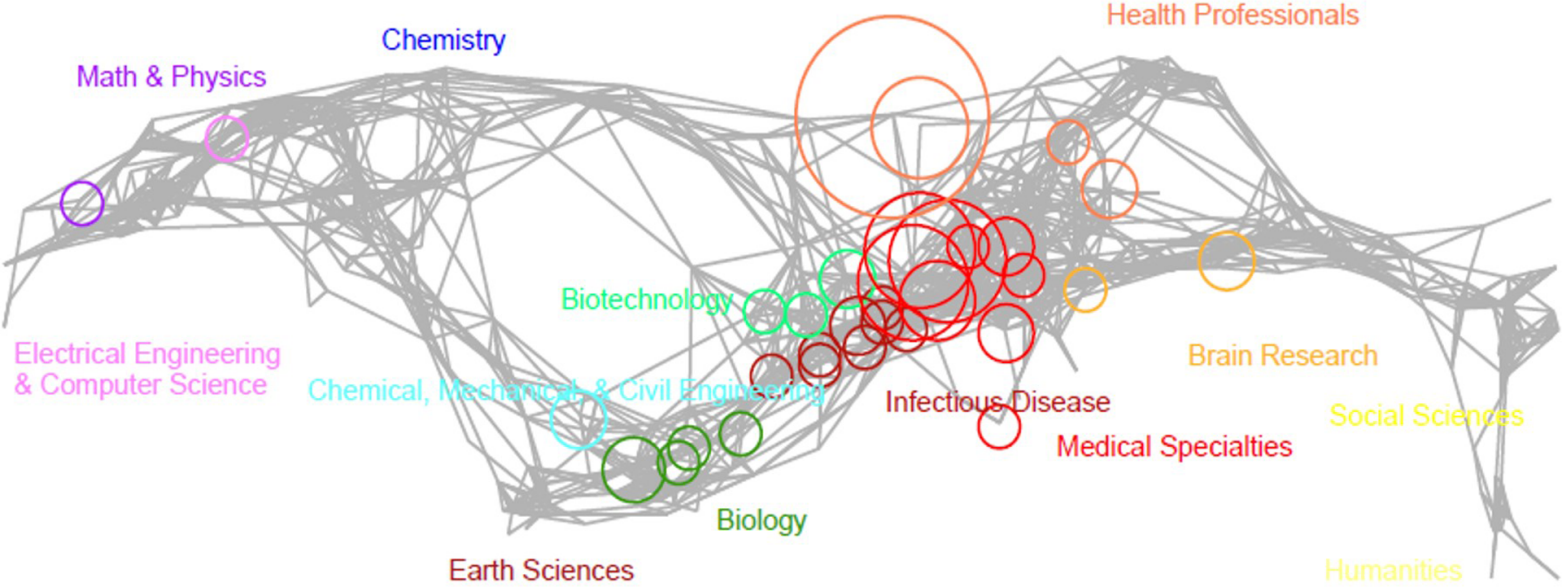
Map of science. It is a visual representation of 554 subdisciplines (nodes) that are aggregated to 13 main disciplines of science. Mapped subdisciplinesare shownby size related to the number matching journals, and colour forthe discipline.

The study of the reproductive effects of EMF exposure on fertility requires a multi-disciplinary approach, for this reason we set up a map representing the co-citation of the papers we identified to study the link among the different disciplines involved in this field (see Fig 5).

As it is evident, unfortunately, hard sciences and electrical engineering on one hand and the health professionals as well as the medical specialities on the other hand, are not so close as it would be desirable, with important difficulties in assuring high quality research and, consequently, reliability of the data with relative inferences.

**Fig 6:**
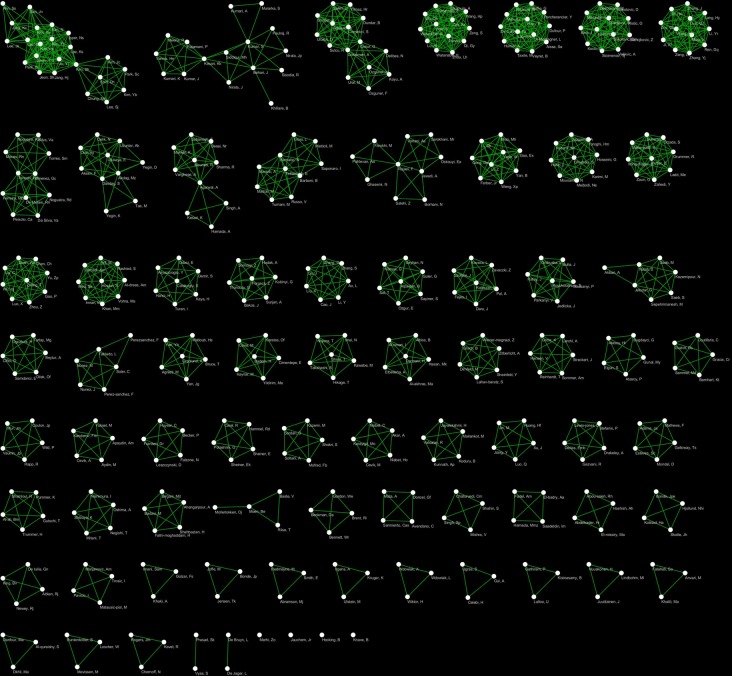
Co_AN. It is a visual representation of Co_AN, in which the Authors are represented as nodes and the co-authorships as links.

Finally, to complete our analysis with the description of authors’ co-authorship dynamics, we set up and analysed co-authorship network (Co_AN) (Table 3 and Fig 6).

**Table 3:** Results of co-authorship network topological analysis.

Parameter	Co_AN	MC1_Co_AN	MC2_Co_AN	MC3_Co_AN
**Number of nodes**	452	23	16	15
**Number of edges**	1339	124	36	65
**Connected components**	74	1	1	1
**Clustering coefficient**	0.953	0.909	0.819	0.941
**Diameter**	3	3	3	2
**Charact. path length**	1.241	1.605	1.967	1.381
**Avg. number of neighbours**	5.925	10.783	4.5	8.667
**γ**		0.163	0.110	-1.112
**r**		0.004	0.112	0.403
**R**^**2**^		0.012	0.022	0.447

For the explanation of topological parameters, refer to the Materials and Methods section.Co_AN = Co-Authorship Network, MC1_Co_AN1, 2, and 3 = Main-Component- Co-Authorship Network1, 2 and 3, are the three larger subnetworks of Co_AN.

In the network, the Authors are represented as nodes and the co-authorship as a link. The analysis of network topology shows that a high number of small size (Main-Components-Co-Authorship Network)connected components (sub-networks) constitutes it: the larger one accounts for about 5% of Co_AN. In addition, all the components are characterized by the tendency to form highly clustered structures that do not communicate with each other. These data suggest that the scientific community involved in the study of such important fields is highly fragmented; highlighting once again the lack of communication among the scientists involved in this such important field. This pattern is specific and different from the researcher network involved in strictly related fields, such as reproductive medicine [21,22], and denotes an important problem in assuring high quality research. Indeed, now it is clear that EMFs act as cofactors with other etiological agents and the related risk is near to the background. Thus we would need big collaborations and transnational networks of researchers to collect a sufficient amount of data [39], otherwise it will be impossible to answer the question on possible negative effects of EMFs on fertility and on heath. Unfortunately, the community involved seems not to have reached an adequate critical mass.

### Conclusions

The study of the possible effects of EMF exposure is an issue of continuously growing importance, in a modern technological society. The bibliometric analysis we carried out leads us to make interesting conclusions. In particular, it is evident that:

- The scientific effort in studying this topic is very limited;
- There are large difference in the research outcome among the different regions and countries, likely due to different research funding but, also, to the different cultural and scientific traditions;
- It would be important to make a larger effort to increase communication among the different researchers involved, with a wide range of competences, from general medicine and assisted reproductive technology, to electrical and electronic engineering, computational dosimetry and the networking activity of Authors.

We think that analysis results could be very interesting for researchers and professionals involved in fertility study (physicians, andrologists, gynaecologists, biologists, embryologists, veterinarians), for clinicians, editors of scientific Journals, as well as editorial board members. In addition, this information could be of interest to officers of funding agencies and of policymaking organisms, as well as for all the people that are interested in carrying out a critical reflection on the effects of EMFs on human and animal health. Indeed, we are faced with new technologies (home automation, smart cities, self-driving cars) that will certainly determine an increase in human exposure to EMFs in the whole environment.

From our data, it is evident that, to obtain reliable information on this topic, it would be necessary to revise of research activity that would be more organized in terms of collaboration and information exchange, with the adoption of standardized models (cellular, animal and exposure parameters) and the realization of large size studies. Research funding could act as a catalyser, to reach these objectives. Unfortunately, to date, the most important programs for research funding, at least in the EU, do not seem to take this opportunity.
